# Design, Simulation, and Fabrication of Multilayer Al_2_O_3_ Ceramic Micro-Hotplates for High Temperature Gas Sensors

**DOI:** 10.3390/s22186778

**Published:** 2022-09-08

**Authors:** Bolun Tang, Yunbo Shi, Jianwei Li, Jie Tang, Qiaohua Feng

**Affiliations:** 1 Higher Educational Key Laboratory for Measuring & Control Technology and Instrumentations of Heilongjiang Province, Harbin University of Science and Technology, Harbin 150080, China; 2 Computer Vision and Intellisense Lab, School of Computer Science, University of Nottingham Ningbo China (UNNC), 199 Taikang East Road, Ningbo 315100, China

**Keywords:** finite element simulation, thick-film gas sensors, microelectromechanical (MEMS) sensors, micro-hotplates

## Abstract

In gas sensors composed of semiconductor metal oxides and two-dimensional materials, the gas-sensitive material is deposited or coated on a metallic signal electrode and must be selective and responsive at a specific temperature. The microelectromechanical devices hosting this material must keep it at the correct operating temperature using a micro-hotplate robust to high temperatures. In this study, three hotplate designs were investigated: electrodes arranged on both sides of an AlN substrate, a micro-hotplate buried in an alumina ceramic substrate, and a beam structure formed using laser punching. The last two designs use magnetron-sputtered ultra-thin AlN films to separate the upper Au interdigital electrodes and lower Pt heating resistor in a sandwich-like structure. The temperature distribution is simulated by the Joule heat model, and the third design has better energy consumption performance. This design was fabricated, and the effect of the rough surface of the alumina ceramic on the preparation was addressed. The experimental results show that the micro-hotplate can operate at nearly 700 °C. The micro-hotplate heats to nearly 240 °C in 2.4 s using a power of ~340 mW. This design makes ceramic-based micro-hotplates a more practical alternative to silicon-based micro-hotplates in gas sensors.

## 1. Introduction

The measurement of flammable, explosive, toxic, and hazardous gases have important roles in industrial production and everyday life [1]. Moreover, globally, climate change is closely related to greenhouse gas emissions [2,3]. According to the Paris Climate Accords, the global level of anthropogenic CO_2_ emitted in 2010 should be reduced by approximately 45% by 2030 [4,5]. Gas sensors are widely used in industrial production and everyday life as the easiest and fastest means of detecting gases [6,7]. There are many different principles of gas sensors, among which sensors that use changes in the resistance of gas-sensitive materials as a gas-sensitive mechanism are particularly common [8]. The mechanism of action of these materials determines the operating temperature of the sensor [9,10]. Materials that exhibit the surface conductivity effect (e.g., SnO_2_ and WO_3_) typically operate at 100–400 °C [11,12,13,14], and those that display the bulk conductivity effect (e.g., TiO_2_ and CeO_2_-TiO_2_) typically operate at 400–700 °C [15,16,17,18]. Few-layer 2D materials (e.g., MoS_2_ and graphene) typically operate from room temperature to 200 °C [19,20,21]. Thus, micro-hotplates compatible with a wide range of temperatures can be used for many types of gas sensor applications with gas-sensitive materials [1,22].

In the field of gas sensors, Si-based materials are very commonly used. Because the same processing technology and mature processing equipment employed for Si wafers and integrated circuit chips can be utilized, Si-based microelectromechanical (MEMS) devices can be made very precise. Furthermore, it is possible to develop a micro heating plate that consumes only 50 mW of power when heated to 500 °C [23,24]. Several studies have explored the process of mutual compatibility between MEMS gas sensors and complementary metal oxide semiconductor [25]. However, gas sensors must be capable of operating at high temperatures for long periods of time and withstanding large temperature shocks. Hence, ceramic substrates, especially alumina ceramic substrates, are often considered to be ideal for sensor substrates [26]. Alumina ceramic substrates have good mechanical stability, high degrees of insulation, and good corrosion resistance. Research into the use of ceramic materials as micro-hotplates for gas sensors has therefore emerged. In 2004, Rettig and Moos [27] were the first to propose the use of low temperature co-fired ceramics (LTCCs) as micro-hotplates. The initial designs achieved better maximum temperatures than the Si-based designs but were larger. The following year, Kita and Rettig [28] investigated whether ceramics would be preferable as micro-hotplates for gas sensors and suggested that ceramics are suitable given the cost, time, and reduced investment requirements of manufacturing technology for small batch production. Ceramic hotplates are appropriate choices, especially when there is a large difference in the coefficient of thermal expansion between the gas-sensitive film and substrate [28]. In 2015, Kita et al. investigated some practical issues that should be considered when preparing ceramic substrates for high temperature gas sensors and concluded that alumina substrates could be considered ideal for sensor substrates [29]. Moreover, both Kulhari and Khanna [30] and Kharbanda et al. [31] arranged the heating resistor and interdigital electrodes on the front and back of the LTCC ceramic plate. These studies differ in that [30] considered different heating resistor design options and [31] considered three ceramic substrate thicknesses. In contrast, the robustness of thicker substrates was accounted for in this study by magnetron sputtering insulating thermally conductive films, and a faster response time was obtained thanks to the ultra-thin insulating layer. Zhao et al. designed a cryogenic gas sensor based on aluminum nitride micro-hotplates for nitrogen dioxide detection [32].

To improve upon the existing designs, this paper proposes a three-layer structure based on a ceramic substrate and using an AlN film as an insulating thermal conductivity layer between the heating resistor and interdigital electrodes. By reducing the distance between the heating resistor and gas-sensitive material, the temperature response of the sensor is improved. Further, an ultraviolet laser is utilized to cut through holes in the brittle alumina ceramic to reduce the power consumption.

## 2. Materials and Methods

### 2.1. Design and Simulation

**Figure 1:**
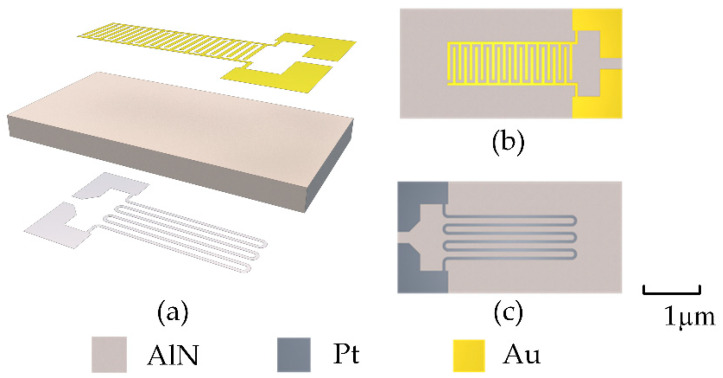
First proposed design using AlN as the substrate. Electrodes are placed on both sides of the substrate. (**a**) Decomposition of the structure, (**b**) front side, and (**c**) back side.

A gas sensor micro-hotplate usually consists of a substrate, heating resistor, and a pair of interdigital electrodes. In this study, we considered three designs. The first uses an AlN ceramic as the substrate, as shown in Figure 1a, with the interdigital electrodes and heating resistor placed on each side of the substrate. This solution is the easiest to achieve in terms of process. The heating resistors distributed on the back of the substrate conduct the heat to the interdigital electrodes located on the front. The substrate is a 4-inch AlN wafer with a thickness of 0.3 mm, which is laser cut after the full process has been completed. The back heating resistor is composed of Pt metal by magnetron sputtering, as shown in Figure 1b, with a thickness of approximately 80 nm, meandered resistive trace with 50 μm width and 100 μm meander spacing. The front interdigital electrodes are composed of Au, prepared by magnetron sputtering, with a thickness of approximately 50 nm, wire width of 50 μm, and wire spacing of 50 μm. The L-shaped structures on both sides are the pads for the leads.

**Figure 2:**
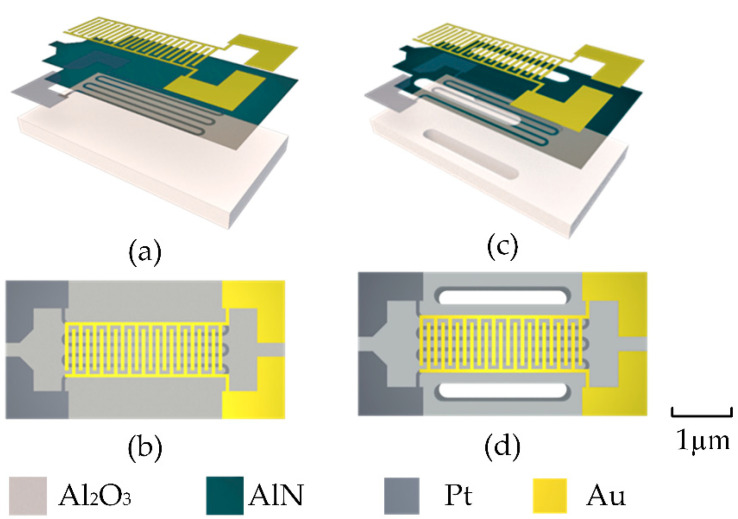
(**a**) Decomposition of the second design, which has an alumina ceramic-based AlN film as the insulating thermal conductivity layer. (**b**) Main view of the second design. (**c**) Decomposition of the third design with the addition of thermal insulation holes. (**d**) Main view of the third design.

The second design option uses a sandwich-like three-layer structure design, as shown in Figure 2a,b, with the electrodes originally arranged on either side of the base on the front. This arrangement brings the heating resistor closer to the interdigital electrodes, which can significantly improve the heating efficiency and reduce the power consumption. To insulate the interdigital electrodes from the heating resistor, we decided to use AlN, which has excellent thermal conductivity, as a spacer layer. The AlN acts as an electrical insulator while conducting the heat from the heating resistor to the upper interdigital electrodes. The thickness of the AlN insulating thermal conductive layer is approximately 500 nm. The choice of an alumina ceramic substrate with good thermal insulation reduces the loss of energy due to temperature conduction to the substrate, thus reducing the power consumption of the sensor. The third design option is based on the second design. To further reduce the diffusion of heat to the sides, we make through-holes 1650 μm in length and 250 μm in width on both sides of the heating plate. The left and right sides outside the through-holes can be clamped or rigidly connected to the outside, as shown in Figure 2c,d.

**Figure 3:**
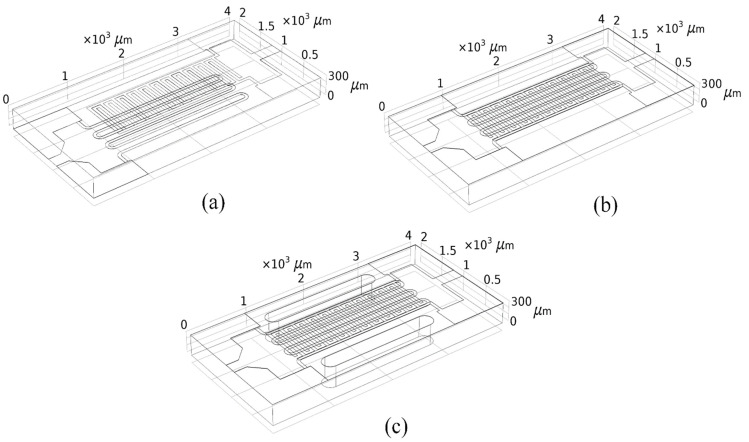
Simulated 3D perspective models of the (**a**) first, (**b**) second, and (**c**) third designs.

The design and simulation of the micro-hotplates described in this paper was performed using COMSOL Multiphysics software. The temperature distribution of the three models at the same voltage was calculated to produce the three three-dimensional (3D) structural models shown in Figure 3. The structural design and simulation analysis of the micro-hotplate are based on the establishment of a heat transfer model, which itself is based on the basic principles of heat transfer.

Heat is transferred by heat conduction, heat convection, and heat radiation. The temperature of the chip increases during operation, and the material parameters of the components change with temperature from the ambient temperature of 20 °C. This behavior makes it impossible to quantify the heat transfer between the components and the heat convection between the chip and environment. The heat transfer from the chip is hence quantified by reducing the dynamic heat transfer process to a steady-state heat transfer. The parameter values used for the material are listed in Table 1.

The initial temperature was set to 20 °C. The two long sides of the micro-hotplate were used to interconnect with the outside world, so the temperature of these two sides was assumed to be 20 °C at all times. Convective heat flux was utilized to calculate the heat transfer between the micro-heating plate and the air, as follows:

(1) $$q_{0}=h\cdot \left(T_{\mathrm{ext}}-T\right)$$

where the conductivity h was set to 5 W/m^2^K and the external temperature T_ext_ was set to 293.15 K. The electro-thermal simulation of the micro-heater was conducted using COMSOL. The distribution of the electric field in solids is governed by the following equation:

(2) −∇⋅(σ⋅∇V)=0

where ∇V is the electric potential, σ is the electric conductivity of the solid, and ∇ is the Laplace operator. The Joule heat Q generated by electric current is determined by:

(3) $$Q=\sigma |\nabla V{|}^{2}$$

The heat transfer in solids is governed by the following equation:

(4) $$\rho_{{\mathrm{vC}}_{p}}\frac{\mathrm{dT}}{\mathrm{dt}}-\nabla \cdot (k\cdot \nabla T)=Q$$

where ∇T is the temperature field, $\rho_{v}$ is the density of solid, $C_{p}$ is the heat capacity at constant pressure, and k is the thermal conductivity of the solids.

### 2.2. Material Suitability

In the design of the MEMS gas sensor micro-hot plate, to satisfy the requirements of sensor miniaturization and low power consumption, the heating resistor should have a low resistance and low power consumption; the interdigital electrodes are usually composed of a stable and corrosion-resistant metal. Simultaneously, the substrate should have a very good temperature uniformity. The difficulty of the process, surface flatness, electrode thickness, and adhesion should all be considered. Au was chosen as the interdigital electrodes in this study because of its resistance to corrosion, low coefficient of thermal expansion, low resistivity, and solderability. Similarly, Pt was chosen for the heating resistor because of its excellent stability and high melting point as well as its relatively stable temperature coefficient of resistance. The choice and design of the substrate material are also particularly important. The substrate must have a certain degree of strength and the ability to cope with rapid temperature changes without deformation. Rapid temperature changes during the use of the sensor may cause thermal expansion of the substrate material, which can easily cause the sensor to break due to the different coefficients of thermal expansion of the different materials in the heating resistor and interdigital electrodes. Two ceramic materials, alumina and AlN, can be employed as substrate materials. The AlN thermal conductivity of 200 W/(m·K) is much higher than that of alumina (24 W/(m·K)). Therefore, in the first design, the AlN substrate was chosen directly, whereas in the latter two designs the more thermally conductive AlN was chosen as the insulating and thermally conductive material between the two layers of electrodes. The properties of the metal materials used in the design are summarized in Table 2.

### 2.3. Manufacturing Method

The micro-hotplates were manufactured mainly via magnetron sputtering, and the devices were made starting from Al_2_O_3_ 100 mm in diameter and 0.3 mm in thickness. A very thin layer of silica was first prepared using low pressure chemical vapor deposition (LPCVD). The treated ceramic substrate was placed in the LPCVD furnace, and the vacuum system was switched on. The main process for LPCVD is as follows: Step 1: Adjust the device to 700 °C. Step 2: Set up gas O_2_/TEOS (Si (OC_2_H_5_)4) flow rate ratio of 1:1.23. Step 3: Set up pressure of 200 mTorr, and precipitation time of 30 min. Annealing was performed at 950 °C for 10 min. After annealing, chemical mechanical polishing was conducted using a polishing pad (IC1000TM, Nitta Haas Inc., Osaka, Japan), polishing solution (Klebosol™ 1501-50, Nitta Haas Inc., Osaka, Japan), platen speed of 90 rpm, carrier speed of 85 rpm, downward pressure of 3–5 psi, and 200 mL/min polishing solution delivery rate.

**Figure 4:**
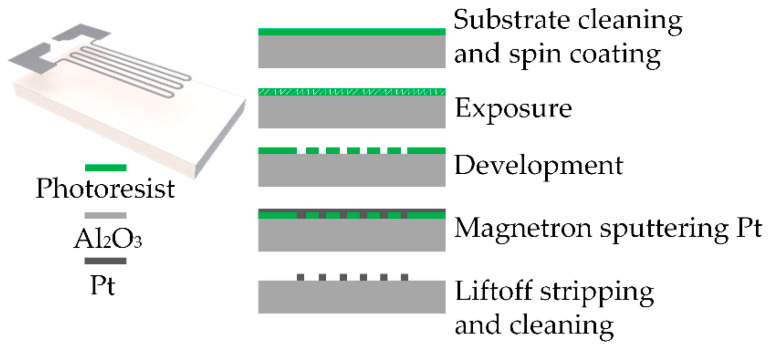
Process flow for the manufacturing of the heating resistor.

The Pt heating resistor, as shown in Figure 4, was fabricated using a lift-off process with no mechanical damage to the substrate during fabrication. The substrate is less susceptible to contamination and the metal film is not subject to lateral drilling; hence, this solution is preferred for magnetron-sputtered metal patterning. A two-step application was performed using a spin-coater machine. The first step was conducted using photoresist (LOR 10A, MicroChem Inc., Newton, MA, USA) as a primer, spin-coating with 3000 rpm for 30 s, and postbaking at 150 °C for 120 s. The second step was conducted using a photoresist (AZ 5214, AZ Electronic Materials Inc., Somerville, NJ, USA), spin-coating with 4000 rpm for 30 s, and postbaking at 95 °C for 90 s. Exposure was performed using double-sided alignment type lithography (MA6, SUSS, Munich, Germany) with an exposure time of 12 s. The exposed substrate was then developed in an RZX-3038 (Suzhou Ruihong Electronic Chemicals Co., Ltd., Suzhou, China) developer for 60–70 s. The developed substrate was placed in a magnetron sputtering machine (JGP-560CRF, Sky Technology Development Co., Ltd., Chinese Academy of Sciences, Shenyang, China), pumped to a high vacuum of 10^−5^ Pa, fed with Ar gas, and sputtered at a power of 200 W for 150 min. The substrate was heated using N-methylpyrrolidone at 120 °C for 150 min. Then, the substrate was cleaned using acetone and deionized water. It was finally annealed by heating at 800 °C for 2 h.

**Figure 5:**
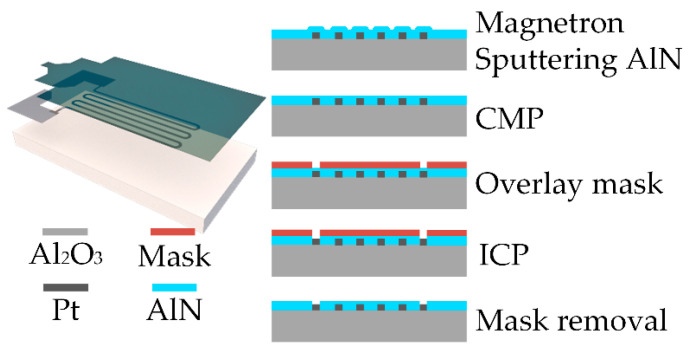
Process flow for the preparation of the AlN insulating and thermally conductive films.

The AlN insulating thermally conductive film was deposited via magnetron sputtering, as shown in Figure 5. The substrate was placed in the magnetron sputtering machine using AlN as the target material, and the distance between the two was adjusted to 80 mm. A vacuum system pressure of 10^−4^ Pa, Ar to N_2_ gas inflow rate of 25:5, and sputtering power of 110 W were used. After sputtering, the substrate was annealed in a vacuum annealing furnace at 100 °C for 120 min. Chemical mechanical polishing was performed to make the AlN raised above the Pt metal completely flat against the surrounding substrate in order to facilitate the formation of the interdigital electrodes using a polishing pad (Suba™ 500, DuPont Co., Ltd., Wilmington, DE, USA) and polishing solution (Nalco^®^ 2370, Nalco Chemical, Chicago, IL, USA). The platen speed was 90 rpm, rotating carrier speed was 85 rpm, downward pressure was 3–5 psi, and flow rate of the polishing solution was 200 mL/min. A mask plate was used to cover the areas other than the heating resistor pads, and inductively coupled plasma etching was carried out using Cl_2_, BCl_3_, and Ar. The inductively coupled plasma coil power was 600 W, pressure was 5 mTorr, bias voltage was −300 V, and etching time was approximately 5–8 min.

**Figure 6:**
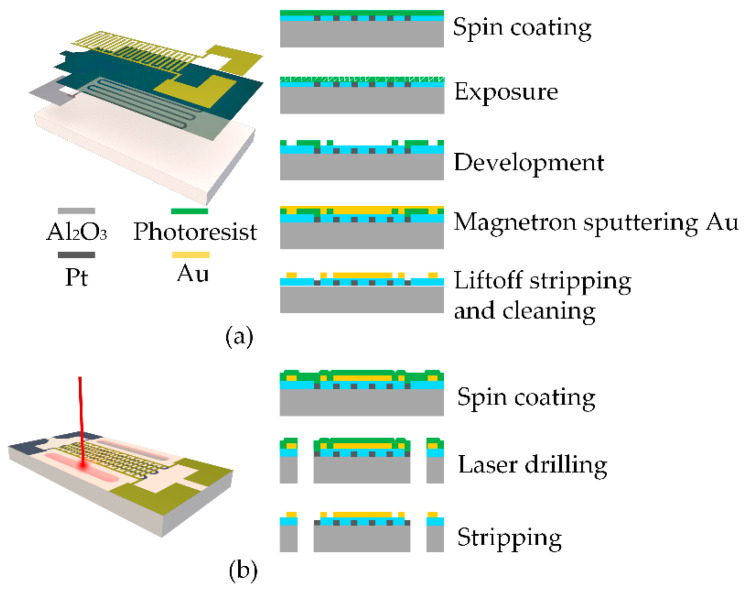
(**a**) Process flow of interdigital electrodes preparation. (**b**) Process flow of laser processing.

The technique used for the Au interdigital electrodes was the same as the magnetron sputtering process utilized for the Pt heating resistor, as shown in Figure 6. Because of the poor adhesion of Au metal on the AlN ceramic, the metal layer of the electrode tends to fall off, so Ti is typically used as the adhesion layer. This is achieved by first sputtering a layer of Ti metal so that the adhesion of the Au is substantially improved, making the surface less likely to fall off. The adhesion layer can improve the adhesion because Ti oxidizes more easily than metals such as Au, Ag, Pt, etc. Therefore, when Ti is deposited on the surface, the very thin metal interface layer is partially oxidized to form covalent bonds. The adhesion layer Ti can diffuse with the upper metal Au, thus creating a strong bond between the metals. Ti bonding layer is 20 nm, and it is fabricated using a magnetron sputtering process. A FOTIA-355 laser with an output wavelength of 355 nm and a pulse width of 11 ns was used. The ultraviolet laser utilized a power of 20 W and scan rate of 20 mm/s. The number of scans was set to 13 to carve through the substrate.

## 3. Results and Discussion

### 3.1. Surface Roughness Optimization

**Figure 7:**
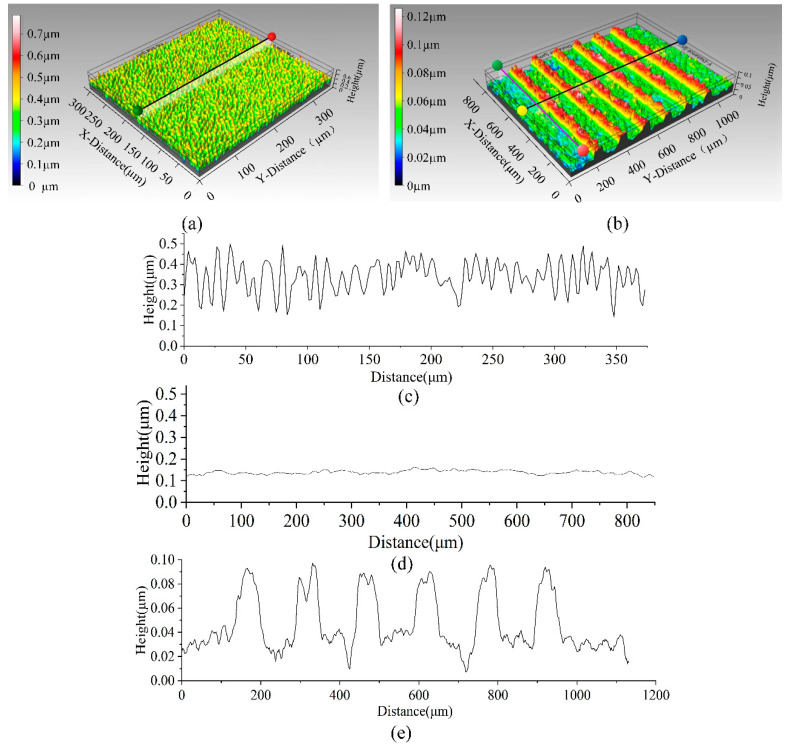
(**a**) Contour of the surface before treatment, (**b**) contour of the heating resistor after preparation, (**c**) height of the section before treatment as indicated by the black line in (**a**), (**d**) height of the section along the purple line in (**b**) after treatment, and (**e**) height diagram along the black line in (**b**) after treatment.

During our initial attempts at fabrication, we found that the approximately 500 nm thickness of the AlN electrical insulation layer did not act as an insulator. This may be caused by the large surface roughness of the alumina substrate and the bulging surface of the substrate piercing the prepared AlN electrical insulation layer. We investigated the surface morphology of the alumina substrate using a profilometer (Contour GT-K 3D, Bruker Daltonics Inc., Billerica, MA, USA), as shown in Figure 7a; the profilometer revealed the presence of peaks and notches on the alumina surface with a maximum height (Sp) of 0.45 μm, a maximum valley depth (Sv) of 0.33 μm, an arithmetic mean height (Sa) of 0.0636 μm, and a root-mean-square height (Sq) of 0.079 μm. Because the thickness of the magnetron sputtered Pt-heating resistor was only approximately 0.1 μm, the surface of the substrate was too rough for to the sputtered Pt electrode film. We therefore prepared a very thin layer of silicon oxide on the substrate surface using LPCVD, followed by chemical mechanical polishing. The thickness of the prepared SiO_2_ is about 2000 nm. This procedure was followed by a magnetron sputtering process for the Pt electrodes, which was characterized using a profilometer on the surface as well as at the electrodes, as shown in Figure 7b. Figure 7c,d show the surface flatness of the substrate along the cut-off line before and after the treatment, respectively. The treatment yielded Sp = 0.029 μm, Sv = 0.030 μm, Sa = 0.0067 μm, and Sq = 0.0084 μm, with an order of magnitude decrease in each value. Figure 7e shows the prepared heating resistor with a thickness of 0.08 μm, width of 50 μm, and pitch of 100 μm, in accordance with the design.

### 3.2. Finite Element Simulation

**Figure 8:**
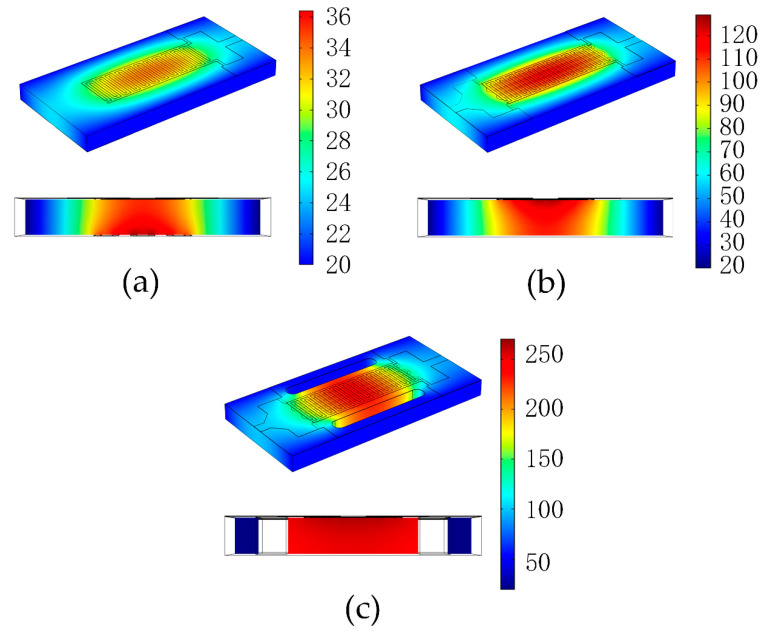
Simulation results of the (**a**) first, (**b**) second, and (**c**) third designs.

The finite element simulation results of the three designs, obtained by applying 10 V to both ends of the heating resistor in each case, are shown in Figure 8. The results for the first design are presented in Figure 8a. The highest temperature occurs in the center of the heating resistor on the back side, whereas the highest temperature in the interdigital electrodes on the front side is only 32 °C. The low temperatures of both the heating resistor and interdigital electrodes are due to the excellent thermal conductivity of the AlN ceramic. The AlN conducts the heat from the heating resistor to the interdigital electrodes and conducts a large amount of heat to the outside world through the contact surfaces on both sides. By contrast, the second sandwich-like three-layer design can reach a maximum temperature of 128 °C, as shown in Figure 8b.

**Figure 9:**
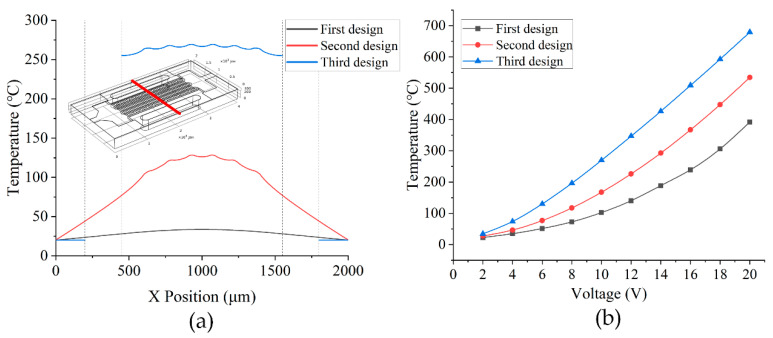
Simulation results for the three designs: (**a**) temperature distribution on the x-axis of the upper surface of the micro-hotplate at a voltage of 10 V and (**b**) variation in the maximum temperature on the upper surface of the micro-hotplate at voltages from 2 V to 20 V.

The third design blocks the transfer of heat to the two surfaces of the micro-hotplate, which are in contact with each other and the outside world through holes on the left and right sides. The cross-sectional view in the simulation shows that the heat released by the heating resistor is mostly concentrated at the location of the interdigital electrodes, and the maximum temperature of the surface can reach 269 °C, which is an increase of 141 °C or 110% compared with the results of the second solution. The temperature data obtained by reading the temperature data along the x-axis at the center of the upper surface of the micro-hotplate in each of the three simulation models are given in Figure 9a. It can be observed that the first design has a very smooth temperature gradient, but the maximum temperature is too small to meet the requirements. The second design has a linear change in temperature gradient, but the third design is clearly superior and has good temperature uniformity in the area of the micro-hotplate, where the sensor material is coated. The breaks in temperature values on either side of the graph are due to the disconnected through-holes, which are designed to isolate the heat loss to some extent. Figure 9b presents the temperature at the highest point as a function of voltage obtained by simulating three designs with voltages between 2 V and 20 V applied in intervals of 2 V.

### 3.3. Temperature Test

**Figure 10:**
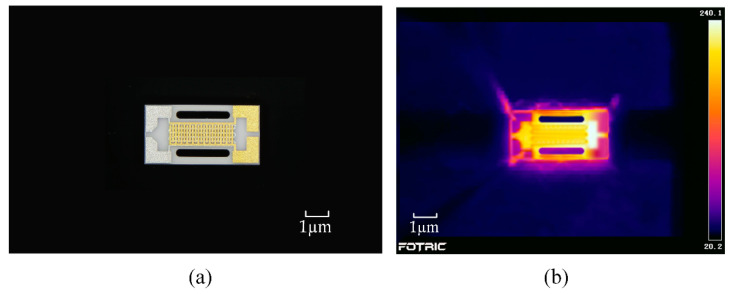
(**a**) Physical view of the completed preparation, (**b**) thermal image of the micro-hotplate, and.

The final micro-hotplate chip, measuring 2 × 4 mm, is shown in Figure 10a. The two sides of the chip are clamped to ensure a secure fixation and the electrodes are connected using Au probes to ensure that higher temperatures can be tested. Figure 10b shows that a maximum temperature of 240 °C is achieved when 10 V is applied to the electrodes using an infrared thermal imager (Fotric 226, Shanghai, China), which agrees with the simulation results.

**Figure 11:**
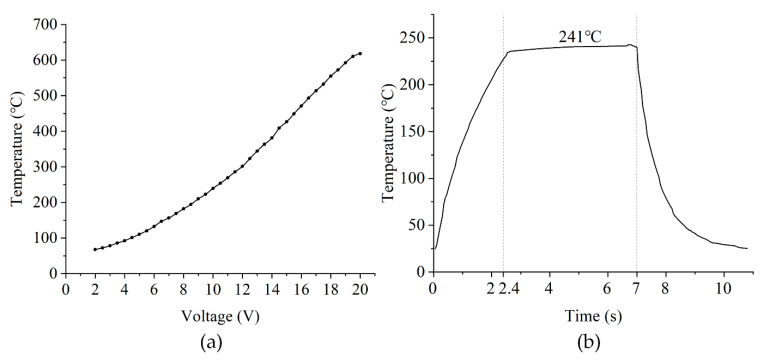
(**a**) Variation in micro-hotplate temperature with respect to input voltage. (**b**) Variation in maximum micro-hotplate temperature with respect to time at 10 V.

Using a programmable power supply (E36312A, Keysight Technologies, Santa Rosa, CA, USA) with 0.5 V steps, heat generation was performed at different voltages ranging from 0.5 V to 20 V, and the temperature was measured with an infrared thermal imager. Figure 11a presents the voltage versus sensor temperature results. The resistance of the heating resistor was measured to be 287.3 Ω. To investigate the response time of the micro-hotplate, the temperature change in the micro-hotplate was obtained at 10 V. The reaction time was defined to be the time to reach 95% of the stable temperature. The results show that the microcalorimetric plate has a response time of 2.4 s during heating and 2.6 s during cooling.

### 3.4. Reliability Assessment

**Figure 12:**
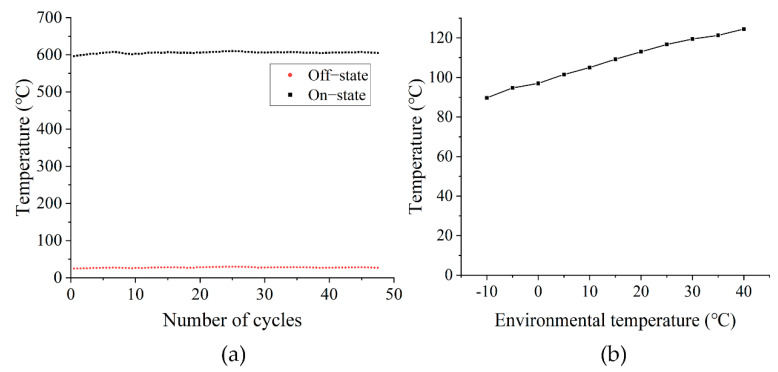
(**a**) Long term on/off cycling test on the fabricated devices. (**b**) The effect of environmental temperature changes on the micro-hot plate.

The micro-hotplate is frequently switched on and off during use, and the temperature usually needs to quickly reach the level suitable for sensitive materials. This frequent switching cycle may damage the micro-hotplate, so we conducted an experiment lasting 9 h per day for 5 days. The switching cycle was 30 min with the micro-hotplate directly reaching the high temperature of approximately 600 °C. During the experiment, no obvious damage to the micro-hotplate and no obvious change in temperature were observed, as shown in Figure 12a.

While using the micro-hotplate, the influence of environmental temperature change cannot be ignored. The influence of the change in the ambient temperature usually needs to be calibrated by the driver circuit and the program. Therefore, we used the high and low temperature alternating test chamber (wdcj-500, China) to simulate the impact of environmental temperature change on the temperature of the micro-hotplate. The temperature was from –10 °C to 40 °C, the voltage of the heating resistor of the micro-hotplate was set to 5 V, and the test was conducted with an interval of 5 °C, as shown in Figure 12b. The influence of ambient temperature on the micro heat plate is significant, but the micro-hotplate has a linear relationship with the change of ambient temperature, which is conducive to the temperature correction in actual use. In the process of manufacturing micro-hotplates, technological tolerances are inevitable. We randomly selected 10 micro-hotplates and measured their temperature values under the same ambient temperature and 10 V at both ends of the heating resistor. The calculated standard deviation of temperature was 1.1898 and the average value was 241.57 °C.

**Figure 13:**
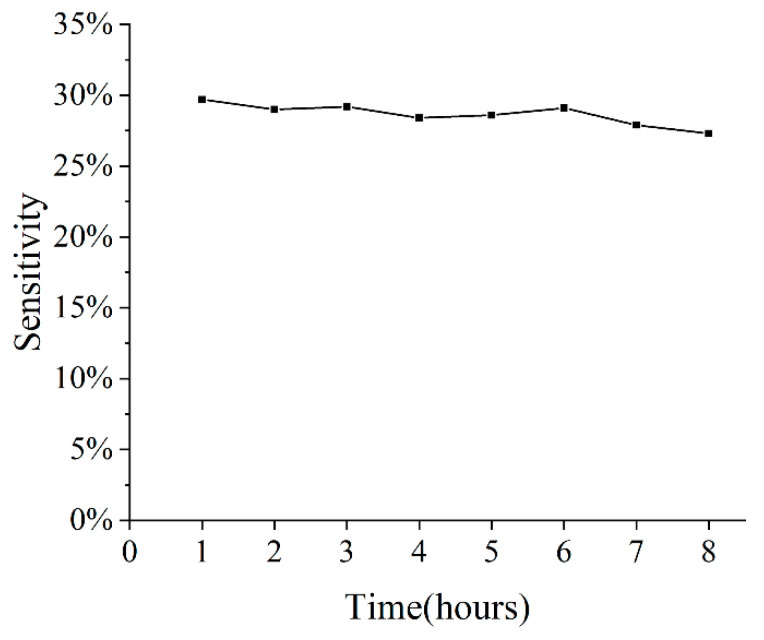
Sensitivity of the SnO_2_ sensor device over time.

The stability of the combination of interdigital electrodes and sensitive materials is also very important. The Au interdigital electrodes are usually stable. We chose the commonly used gas-sensitive material SnO_2_ to preform stability tests. The SnO_2_ was prepared by hydrothermal method, mixed with terpineol to form a slurry, and then coated on the surface of the micro-heater. A 500-ppm ethanol gas was used as the test gas for the stability test over a long time. The sensor device at a constant operating temperature and ethanol gas was pumped into the chamber every one hour. The resistance R_0_ of the sensitive material in the air and the resistance R in the ethanol gas under the working state of the sensor, sensitivity is defined as (R_0_ − R)/R_0_. The sensitivity of the sensor was tested, as shown in Figure 13. Results show that the sensitivity kept stable (variate less than 2%) over 8 h.

### 3.5. Relationship to Existing Designs

Ceramics were first used as micro-hotplates by Rettig and Moos [27] and Kita et al. [28], but the wide line spacing utilized in these designs tended to cause uneven temperature distributions; a structure with cantilevered beams was less strong and stable, but it gave the micro-hotplate excellent power consumption performance [28]. In [30], the size of the micro-hotplate was significantly reduced, and the cantilever beam structure was removed, optimizing the stability of the structure but also increasing the power consumption; further, the performance achievable with various thicknesses was compared, and it was suggested that thinner substrates were beneficial for temperature conduction. Kharbanda et al. [31] were the first to investigate the effects of heating resistor with different wire spacings and concluded that a narrower wire spacing was preferable. Kulhari et al. [33] were the first to propose micro-hotplates with positive temperature coefficient (PTC) sensors using a lamination technique to laminate multiple layers of ceramic plates together to isolate the interdigital electrodes, heating resistors, and PTC electrodes. 

In this paper, an innovative magnetron sputtering process compatible with a Si-based MEMS device was used to isolate the heating resistor and interdigital electrodes using a very thin layer of AlN ceramic, which has excellent thermal conductivity. The beam structure employed in [27] was also used to create thermal insulation holes on both sides of the heating resistor. The relevant parameters of the proposed design are compared with those reported in related literature in Table 3.

## 4. Conclusions

This paper presented the design, simulation, and fabrication of an alumina ceramic-based micro-hotplate for gas sensors, using ultra-thin AlN as the insulating thermally conductive layer. Existing studies have shown that using a thinner alumina ceramic substrate reduces the substrate area while considering the strength. A heating resistor with a line width of 50 μm and line spacing of 100 μm, based on previously reported results, was used to ensure a uniform temperature distribution on the surface of the micro-hotplate. By simulating three different designs, it was concluded that the use of an insulating thermally conductive film can significantly increase the temperature at the same voltage when compared with the direct use of an AlN substrate on both sides. The laser-fabricated insulating holes reduce heat dissipation and increase the temperature while being more robust and stable than a beam-suspended structure. To solve the problem of the rough surface of the alumina ceramic, a layer of silicon oxide was prepared on the surface of the alumina and then chemically and mechanically polished. The final micro-hotplate could reliably achieve temperatures of nearly 700 °C, heat to nearly 300 °C at a power of approximately 480 mW, and achieve a response time of approximately 2.4 s. 

Although this design is an improvement compared to the existing designs of ceramic-based micro-hotplates for gas sensors, it would benefit from improvements in size and response time compared with Si-based micro-hotplates. In addition, with the advancement of MEMS processes, ceramic-based micro-hotplates should be investigated using approaches similar to those of Si-based sacrificial layer and bonding processes, among others, to thin the substrate further or create cavities to improve the thermal efficiency. Currently, two-dimensional materials including graphene and black phosphorus are hot spots for research on gas-sensitive materials; however, a more prominent problem now is how to form gas-sensitive films. Chemical vapor deposition and high-temperature mineralization are the main preparation methods for graphene and black phosphorus. Furthermore, high temperature resistant ceramic micro-hotplates can be placed in chemical vapor deposition equipment to achieve the growth of gas sensitive materials directly on the micro-hotplates. Controlling the film thickness by adjusting the supply of carbon source in the chemical vapor deposition process may be a potential research direction.

## Figures and Tables

**Table 1:** Main material parameters used in the simulation.

Material Property	AlN	Alumina	Au	Pt
Thermal conductivity (W/m·K)	200	20	317	72
Specific heat (J/kg/K)	780	750	129	133
Electrical conductivity (S/m)	<10^−14^	<10^−14^	4.10 × 10^7^	9.43 × 10^6^
Density (kg/cm^3^)	3100	3600	1.932 × 10^4^	2.145 × 10^4^

**Table 2:** Properties of metal materials.

Material Property	Pt	Au	Ag	Cu	Al
Resistivity (μΩ·cm)	22.7	2.26	1.62	1.71	2.71
Coefficient of thermal expansion (10^−6^ K^−1^)	8.8	14.2	18.9	16.5	23.1
Melting point (°C)	2041	1337	1235	1358	933
Solderability	Easy	Easy	Easy	Easy	Difficult
Air stability	Stable	Stable	Generally stable	Unstable	Stable

**Table 3:** Comparison of different ceramic micro-hotplate designs.

Material Property	Rettig and Moos [25]	Kulhari and Khanna [28]	Kharbanda et al. [29]	Kulhari et al. [30]	Proposed Design
Size (mm)	10 × 10	3.4 × 3.4	2.5 × 2.5	8 × 8	4 × 2
Line width (μm)	300–500	200	50	~200	50
Line spacing (μm)	200–400	200	260/100	~200	100
Insulation layer thickness (mm)	0.4	0.711	0.7	--	0.002
Power consumption (mW)	598 (400 °C)	1450 (468 °C)	500 (300 °C)	850 (300 °C)	480 (300 °C)
Response time (s)	--	12 (280 °C)	20 (340 °C)	--	2.4 (240 °C)
